# The expression and clinical prognostic value of protein phosphatase 1 catalytic subunit beta in pancreatic cancer

**DOI:** 10.1080/21655979.2021.1934243

**Published:** 2021-06-14

**Authors:** Lingyu Hu, Haokai Xu, Xiaoguang Wang, Bin Wu, Fei Chen, Wei Chen, Yong Gao, Zhengxiang Zhong

**Affiliations:** a Bengbu Medical College, Bengbu, Anhui P.R. China; b Department of Surgery, The Second Affiliated Hospital of Jiaxing University, Jiaxing, Zhejiang P.R. China; c Department of Surgery, Ningbo Yinzhou No.2 Hospital, Ningbo, Zhejiang, P. R. China; d Cancer Institute of Integrated Traditional Chinese and Western Medicine, Zhejiang Academy of Traditional Chinese Medicine, Tongde Hospital of Zhejiang Province, Hangzhou, Zhejiang, P.R. China

**Keywords:** Bioinformatics analysis, protein phosphatase 1 catalytic subunit beta, pancreatic adenocarcinoma, prognosis

## Abstract

Pancreatic cancer (PAAD) is a common malignancy with a poor survival rate. The identification of novel biomarkers could improve clinical outcomes for patients with PAAD. Here we evaluated the expression and clinical significance of *PPP1CB* in PAAD. *PPP1CB* expression was higher in PAAD tissue than in matched paracancerous tissue (*P < 0.05*). We predicted a network of regulatory targets and protein interaction partners of *PPP1CB*, and identified a PPI network consisting of 39 node genes. The expression of 33 node genes was higher in PAAD tissue than in matching paracancerous tissue. High expression of the node genes *ACTN4, ANLN, CLTB, IQGAP1, SPTAN1*, and *TMOD3* was associated with improved overall survival (*P < 0.05*). SiRNA knockdown of *PPP1CB* significantly reduced the migration and invasion of PAAD cells. A *PPP1CB* immunohistochemical staining was performed using a tissue microarray (TMA), consisting of tumor samples collected from 91 patients with PAAD (88 of which contained matched paracancerous tissues). The expression of *PPP1CB* in PAAD was significantly higher than in the matched paracancerous tissue, (*P = 0.016*). High *PPP1CB* expression was associated with patient sex (*P = 0.048*), alcohol use (*P = 0.039*), CEA **(***P**= 0.038***), N stage **(***P = 0.001*), and invasion of nerve (*P = 0.036*). Furthermore, high *PPP1CB* expression was associated with significantly poorer overall survival (*P = 0.022*). Our data demonstrate that *PPP1CB* is associated with the migration and invasion of PAAD cells, and may be useful as an independent prognostic indicator for clinical outcome in patients with PAAD.

## Graphical abstract

**Figure:**
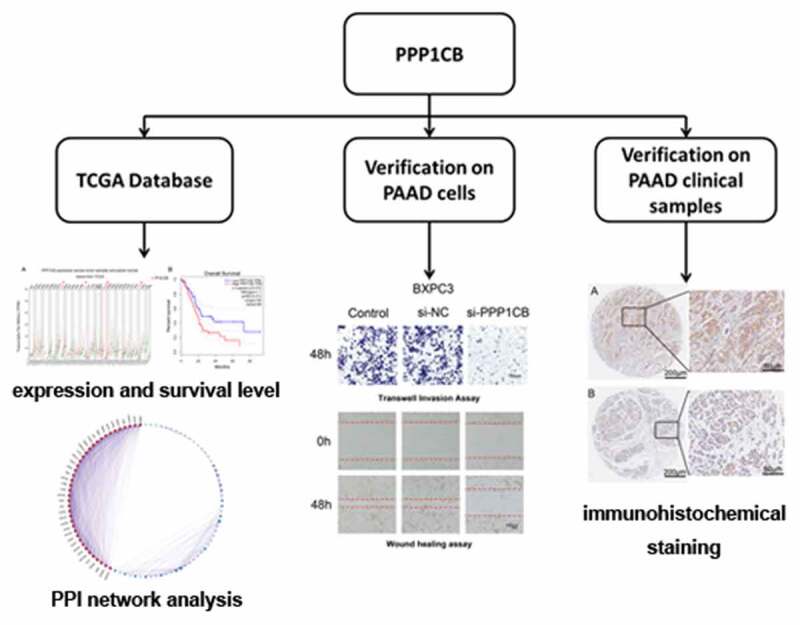

## Introduction

Pancreatic cancer (PAAD) is a highly malignant and fatal tumor. Despite treatment advances, the 5-year survival rate of PAAD remains at only 8%, and PAAD is expected to become the second greatest cause of cancer death over the next two decades [1,2]. Approximately 80–85% of patients with PAAD will have advanced metastases that cannot be surgically removed [3]. Unfortunately, conventional radiotherapy and chemotherapy treatments do not significantly prolong the survival of PAAD patients [4,5]. Even in the cases of those patients who are diagnosed with locally resectable tumors, disease metastasis and recurrence frequently occur after surgery, and the prognosis remains dismal [5]. There is a need to estimate the survival rate of patients after operation for resectable PAAD, but the current prognostic methods are inaccurate [6].

As one of the catalytic subunits of protein phosphatase 1 (*PP1C*), protein phosphatase 1 catalytic subunit beta (*PPP1CB*; encoded by *PPP1CB gene*) is a serine/threonine-specific protein phosphatase, and is involved in the regulation of a variety of cellular processes such as survival, cell cycle regulation, and apoptosis [7]. *PPP1CB* is important for the dephosphorylation of myosin light chain [8]. Phosphorylation of a *PPP1CB* inhibitor can promote contractile actomyosin mechanisms and accelerate tumor metastasis [9]. *PPP1CB* plays a vital role in regulating the cytoskeleton network and cell migration, processes germane to cancer invasion and metastasis [10]. The absence of *PPP1CB* negatively impacts nuclear integrity, resulting in nuclear fragmentation, nuclear envelope rupture, loss of the nuclear compartment barrier, and genome instability [11,12]. *PPP1CB* is also associated with metastasis in gastric cancer and melanoma, and is a latent anti-metastatic target for the treatment of early stage melanoma [13,14]. As a regulator of endothelial cell migration, *PPP1CB* may be an effective target for anti-angiogenic therapy and inhibition of tumor growth [15]. Additionally, *PPP1CA, PPP1CB, PPP2CA, PPP2CB*, and *PPP3CA* facilitate PAAD progression, while *PPP3CB, PPP5C*, and *PPP6C* inhibit PAAD progression, however the role of *PPP1CB* has not been thoroughly investigated [16].

Therefore, we evaluated the correlation between *PPP1CB* expression and PAAD prognosis in this study, and we explored the role of *PPP1CB* role in PAAD progression.

We analyzed gene expression from The Cancer Genome Atlas (TCGA) public dataset to evaluate *PPP1CB* expression in PAAD, to screen for potential *PPP1CB* interaction partners, and to examine expression correlation between *PPP1CB* and potential interacting partners via protein-protein interaction (PPI), and to explore the prognostic value of these genes on clinical outcomes for PAAD patients.

Based on previous findings, we speculated that *PPP1CB* may serve as an independent predictor of poor PAAD outcome. In this study, the effects of *PPP1CB* knockdown on the migration and invasion of PAAD cells was evaluated. We investigated the expression levels of *PPP1CB* in tumor samples from PAAD patients and determined the correlation of *PPP1CB* expression with clinical outcomes of PAAD patients.

## Materials and methods

### Bioinformatics analysis

#### PPP1CB expression and prediction of interacting protein partners

TCGA is a multidimensional human cancer dataset, including gene expression profiling and clinical data [17]. We obtained the gene expression of *PPP1CB* from a variety of human cancers and matched with paracancerous tissues, Table I shows the types of cancers and the number of cases assessed by the TCGA dataset. (Table I), and used the GEPIA tool (http://gepia.cancer-pku.cn/) to analyze these data [18]. Based on the UCSC Xena project (http://xena.ucsc.edu), the TCGA dataset GEPIA tool was used to determine the optimal sample size to compare tumors with the matched paracancerous non-tumor tissue [19].

**Table I.** Cancer types evaluated from the TCGA dataset

Types of cancer	TCGA dataset	No. of cancer tissues	No. of normal tissues
Adrenocortical carcinoma	TCGA-ACC	77	128
Bladder Urothelial Carcinoma	TCGA-BLCA	404	28
Breast invasive carcinoma	TCGA-BRCA	1085	291
Cervical squamous cell carcinoma and endocervical adenocarcinoma	TCGA-CESC	306	13
Cholangio carcinoma	TCGA-CHOL	36	9
Colon adenocarcinoma	TCGA-COAD	275	349
Lymphoid Neoplasm Diffuse Large B-cell Lymphoma	TCGA-DLBC	47	337
Esophageal carcinoma	TCGA-ESCA	182	286
Glioblastoma multiforme	TCGA-GBM	163	207
Head and Neck squamous cell carcinoma	TCGA-HNSC	519	44
Kidney Chromophobe	TCGA-KICH	66	53
Kidney renal clear cell carcinoma	TCGA-KIRC	523	100
Kidney renal papillary cell carcinoma	TCGA-KIRP	286	60
Acute Myeloid Leukemia	TCGA-LAML	173	70
Brain Lower Grade Glioma	TCGA-LGG	518	207
Liver hepatocellular carcinoma	TCGA-LIHC	369	160
Lung adenocarcinoma	TCGA-LUAD	483	347
Lung squamous cell carcinoma	TCGA-LUSC	486	338
Mesothelioma	TCGA-MESO	87	0
Ovarian serous cystadenocarcinoma	TCGA-OV	426	88
Pancreatic adenocarcinoma	TCGA-PAAD	179	171
Pheochromocytoma and Paraganglioma	TCGA-PCPG	182	3
Prostate adenocarcinoma	TCGA-PRAD	492	152
Rectum adenocarcinoma	TCGA-PEAD	92	318
Sarcoma	TCGA-SARC	262	2
Skin Cutaneous Melanoma	TCGA-SKCM	461	558
Stomach adenocarcinoma	TCGA-STAD	408	211
Testicular Germ Cell Tumors	TCGA-TGCT	137	165
Thyroid carcinoma	TCGA-THCA	512	337
Thymoma	TCGA-THYM	118	339
Uterine Corpus Endometrial Carcinoma	TCGA-UCEC	174	91
Uterine Carcinosarcoma	TCGA-UCS	57	78

The term ‘PPPC1B’ was entered into the following databases to predict protein interactions and target genes regulated by *PPPCIB*: STRINGv11.0 (https://string-db.org/), InBioMapv2019 (https://www.intomics.com/inbio/map/#home), BioGRID v4.0 (https://thebiogrid.org/), HPRD v2009 (http://www.hprd.org/), and IntAct v4.2.16 (https://www.ebi.ac.uk/intact/). Screening criteria for potential interacting partners were determined based on the optimal homology score for each gene database [20–25]. We constructed a Venn diagram to further screen the prediction results and selected overlapping predicted genes for further in-depth analysis (http://bioinformatics.psb.ugent.be/webtools/Venn/).

#### Enrichment analysis and PPI network analysis in Metascape

Metascape version 2019 (http://metascape.org/gp/index.html) was used for enrichment analysis of the overlapping *PPP1CB* predicted interacting partners [26]. CORUM, GO, KEGG Pathway, and Reactome Gene Sets were constructed to determine the biological functions related to the target genes. The overlapping interacting partners of *PPP1CB* were analyzed by a protein-protein interaction (PPI) network using Metascape. Node scores were calculated in Metascape, and the cutoff value of node scores was determined to be 1.273. The module Molecular Complex Detection (MCODE) algorithm was used to generate intensively connected networks, and different MCODE components are indicated by color coding to represent potential biological significance [27].

#### Analysis of the target gene correlation with disease prognosis

The gene expression profile interactive analysis (GEPIA) (http://gepia.cancer-pku.cn/) tool was used to analyze the node target genes, and to compare the expression levels of these genes in tumors with matched paracancerous tissues, in order to determine the prognostic value of these genes in predicting PAAD outcomes [18].

### Verification experiments on PAAD cells

#### Cell source and culture

Human PAAD cell lines (BXPC3, CAPAN-1, PANC-1) were acquired from the American Type Culture Collection (Manassas, Virginia, USA). BXPC3 and CAPAN-1 cells were cultured in Roswell Park Memorial Institute (RPMI) 1640 medium (Gibco, Grand Island, New York, USA), and PANC-1 cells were cultured in Dulbecco’s Modified Eagle’s Medium (DMEM; Gibco, Carlsbad, California, USA). All culture medium was supplemented with 10% fetal bovine serum (FBS; Gibco, Grand Island, New York, USA) and 1% penicillin/streptomycin (Sigma, St. Louis, Missouri, USA). All cells were cultured at 37°C in a 5% CO_2_ incubator. The same culture medium was used for subcultures. **The cell lines were passaged at a dilution of 1:3–1:4 every 3–5 days.**

#### RNA Transfection

Small interfering RNA (siRNA) targeting *PPP1CB* was purchased from Genepharma Biotechnology (Shanghai, China) and Lipofectamine-2000 (Invitrogen, Carlsbad, California, USA) was used to transfect PAAD cells with the siRNA, in accordance with the manufacturer’s instructions. The siRNA sequences are as follows: *PPP1CB*-homo-459: 5'- GCAGAAGUUCGAGGCUUAUTT-3' and 5'- AUAAGCCUCGAACUUCUGCTT-3'; *PPP1CB*-homo-911: 5'- GGAGCAGAUUCGGAGAAUUTT-3' and 5'- AAUUCUCCGAAUCUGCUCCTT-3'; *PPP1CB*-homo-1137: 5'- GCUAAACGACAGUUGGUAATT-3' and 5'- UUACCAACUGUCGUUUAGCTT-3'; Negative Control: 5'-UUCUCCGAACGUGUCACGUTT-3' and 5'-ACGUGACACGUUCGGAGAATT-3'.

#### Western blotting

**C**ell lysis buffer (Cell Signaling Technology, Danfoss, Massachusetts, USA) was used to extract proteins from PAAD cells. Protein concentration was determined using a bicinchoninic acid protein assay kit (BCA; Sigma-Aldrich, Merck KGaA, Darmstadt, Germany). Then the protein samples (40 μg/lane) were separated by 10% sodium dodecyl sulfate-polyacrylamide gel electrophoresis (SDS PAGE), and transferred to polyvinylidene fluoride (PVDF) membranes (Millipore, Billerica, Massachusetts, USA). Tris buffered saline and 0.1% Tween 20 (TBST) with 5% bovine serum albumin (BSA) was used to block the membranes. Subsequently, membranes were incubated with primary antibody against *PPP1CB* (1:1000 dilution in TBST; Abcam, Cambridge, Massachusetts, USA) overnight at 4°C. After rinsing with 0.1% TBST three times, membranes were incubated with HRP-labeled secondary antibodies (1:2000 dilution in TBST; Abcam, Cambridge, Massachusetts, USA) of the corresponding species at room temperature for 2 hours with gentle shaking. The normalization control used was α-tubulin protein expression. The immunoreacted proteins were detected using an Enhanced Chemiluminescence Detection Kit-HRP (EZ-ECL, 20–500-120, Biological Industries, ISR), and the level of protein expression was determined.

#### Wound healing assay

A wound healing assay was used to study the impact of *PPP1CB* knockdown on PAAD cell migration. Briefly, cells were seeded at 3 × 10^5^ cells/well in 6-well plates, and then transfected with si-*PPP1CB* or si-NC. A micropipette tip was used to create a wound in a cell monolayer. The wound was washed with PBS, and photographed at 0 hours and 48 hours under an inverted optical microscope (Olympus IX51, Olympus, Center Valley, Pennsylvania, USA). The ratio of the residual wound area to the initial area was calculated and quantified by ImagePro Plus V 6.0 (Media Cybernetics, Bethesda, Maryland, USA).

#### Transwell invasion assay

Cell invasion was examined using a 24-well Transwell chamber (8 μm; Corning, New York, USA). First, PAAD cells (5 × 10^4^ cell) were seeded into the upper chamber in 200 μL medium without FBS, and 700 μL complete medium (containing 10% FBS) was added to the bottom chamber. After incubation for 48 hours, the cells that had invaded to the bottom of the Transwell insert were washed with PBS, fixed with methanol for 10 minutes, and then stained with 0.1% crystal violet for 10 minutes. Using a reverse microscope (Olympus, Tokyo, Japan) at 40× magnification, the cell membrane was observed in five random fields, and the number of cells that had migrated was counted and photographed.

### Verification experiments on PAAD clinical samples

#### Clinical samples collection

A PAAD tissue microarray (TMA) was acquired from the National Human Genetic Resources Sharing Service Platform (Number: 2005DKA21300). The experimental procedures were approved by the Shanghai Outdo Biotech Company ethics committee (Number: YB M-05-02), which authorized the collection of tissue samples from patients. The TMA included 91 PAAD tumor tissues, 88 of which also included matched paracancerous tissues. All patients signed informed consent, these patients underwent resection surgery between January 2004 and December 2013. Clinicopathological information included age (year), N stage, histology, invasion of nerve, invasion of large blood vessels, jaundice status, alcohol use, M stage, location, T stage, CA199, grade, CEA, tumor sizes, TopoII, sex, diabetes, smoking, TNM stage, and p53, p63, and Ki67 status. In total, 88 pairs of cancer tissue and matched paracancerous tissue were used to analyze the expression differences between cancer and matched paracancerous tissue. For the correlation analysis relationships between clinicopathological information and PAAD gene expression, all 91 PAAD tissue samples were used, even though about three samples did not have matched paracancerous tissues.

#### Immunohistochemical staining evaluation

Immunohistochemical staining of the PAAD TMA was performed using an immunohistochemistry kit (EnVision™ FLEX+, cat. no. K8002, Dako; Agilent Technologies, Inc.). The Biochip Shanghai National Human Genetic Resources Sharing Service Platform provides an Automated Autostainer Link 48 system (Dako; Agilent Technologies, Inc.). In order to evaluate the expression of *PPP1CB* in tissue samples, two professional pathologists used an Aperio scanner (Aperio XT, Leica Microsystems GmbH, magnification, x200) to score immunohistochemical staining. The intensity of cytoplasm/nucleus staining and the positive staining rate of *PPP1CB* in PAAD and matched paracancerous tissues were measured, and the cytoplasm and nuclear staining were scored, respectively. Staining intensity scoring standards were as follows: 0 points (negative), 1 point (+), 2 points (++), 3 points (+++). A revised score is given to the staining intensity of each sample based on criteria [26]. Finally, the product of ‘staining intensity scores’ and ‘staining rate’ is the total score for the group. Samples with a score **<**1.2 were categorized into the low expression group, and samples ≥1.2 were categorized into the high expression group.

## Statistical analysis

Fisher’s exact test and Chi-square test were used to analyze the expression of *PPP1CB* in PAAD specimens and matched paracancerous tissues, as well to assess relationship between *PPP1CB* expression and clinicopathological characteristics. The Kaplan‑Meier method was used to create survival curves, and univariate survival analysis utilized the log-rank statistical test. Statistical analyses were performed using SPSS version 22.0 (SPSS, Inc.) and GraphPad Prism version 8 (GraphPad, San Diego, California, USA). A P-value of *P < 0.05* was considered to be statistically significant.

## Results

### Bioinformatics analysis

#### PPP1CB expression in the TCGA database

**Figure 1.**
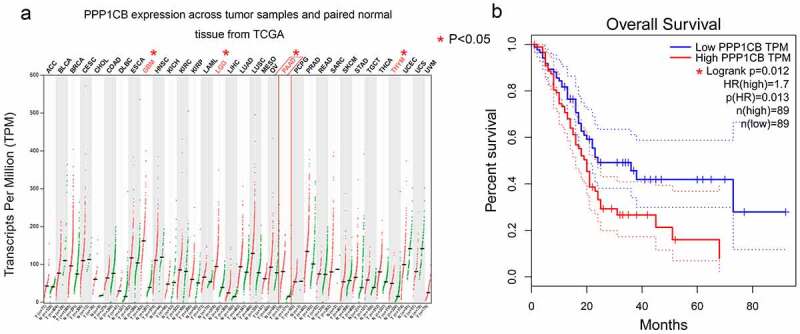
*PPP1CB* expression profiling in cancer tissue and normal tissue from The Cancer Genome Atlas. (a) *PPP1CB* expression across tumor datasets (T; red dots) and matched paracancerous tissue (N; green dots) datasets. Each single point represents the expression of *PPP1CB* in a single sample. Comparison between tumor and normal tissue was performed using the GEPIA tool, and significantly elevated expression was determined by a high log2FC value and a percentage value greater than the threshold value. Cancer types indicated in red have significantly higher *PPP1CB* expression than corresponding normal tissue. (b) Kaplan‑Meier survival analysis of patients based on *PPP1CB* expression (data from TCGA datasets). HR: hazard ratio. *P < 0.05.

In order to investigate the expression of *PPP1CB* in pancreatic cancer and its clinical prognostic value, we first performed a bioinformatics analysis. *PPP1CB* is highly expressed in a variety of human cancers, including pancreatic adenocarcinoma (PAAD), glioblastoma multiforme (GBM), thymoma (THYM), and brain lower grade glioma (LGG) (*P < 0.05*), compared to normal tissue. *PPP1CB* was not differentially expressed between normal and cancer tissue in other cancers, such as adrenocortical carcinoma (ACC), lung adenocarcinoma (LUAD), or prostate adenocarcinoma (PRAD). These results demonstrated that *PPP1CB* is significantly differentially expressed in various tumors compared to normal tissue, including significantly elevated expression in PAAD, suggesting that *PPP1CB* might play a role as an oncogene (Figure 1a).

#### High PPP1CB expression is associated with poor prognosis in patients with PAAD

We analyzed data from 178 patients in the TCGA dataset. These patients were subset into a low *PPP1CB* expression group (n = 89), and a high *PPP1CB* expression group (n = 89). Low expression of *PPP1CB* was significantly associated with improved survival (*P = 0.012*; Figure 1b).

#### Identification of PPP1CB interaction partners/regulatory target genes

**Figure 2.**
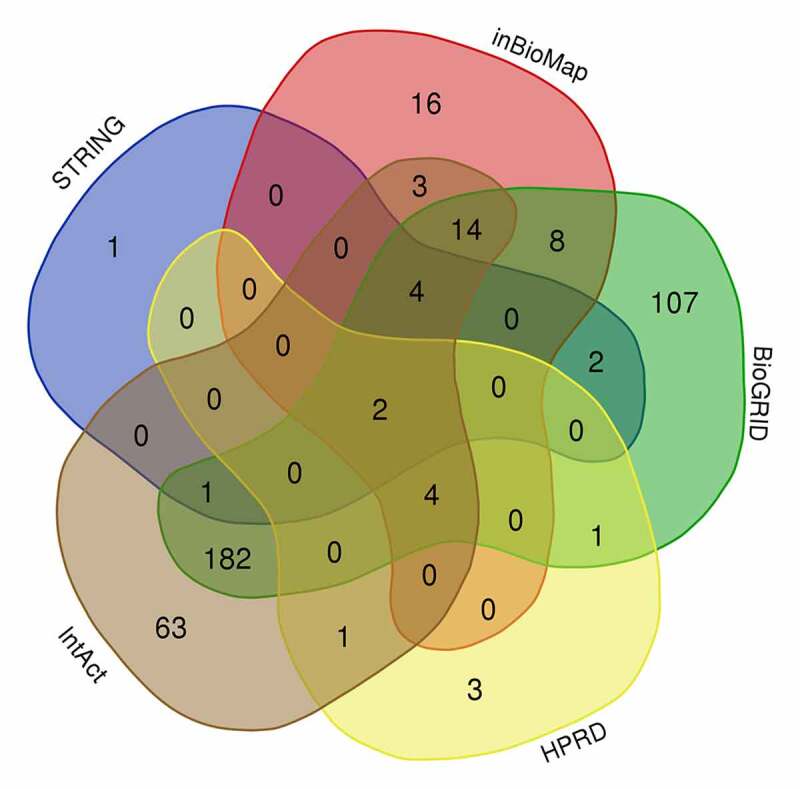
The Venn diagram produced by five databases to predict *PPP1CB* interacting partners and regulatory target genes. Each color represents the corresponding database

Using InBioMap, STRING, BioGRID, HPRD and IntAct, we identified 51, 10, 325, 11, and 273 *PPP1CB* candidate interaction partners/target genes, respectively (Supplementary file 1). Moreover, 222 genes from these five databases overlapped in at least two databases, shown in the Venn diagram **(**Figure 2**). T**hese overlapping genes were selected for further analysis.

#### Enrichment analysis of candidate PPP1CB interacting partners

The biological processes and functions of 222 target genes through pathway enrichment analysis were explored. Enrichment analysis demonstrated that the target genes of *PPP1CB* take part in GO Biological Processes, including ‘regulation of dephosphorylation’, ‘actin filament-based movement’, ‘positive regulation of organelle organization’, ‘actomyosin structure organization’, ‘cell morphogenesis involved in differentiation’, ‘actin cytoskeleton organization’, ‘cell morphogenesis involved in differentiation’, ‘cell division’, ‘cell junction assembly’, ‘actin filament bundle assembly’, and ‘actin filament severing’. KEGG pathway analysis identified enrichment of target genes in the following KEGG Pathways: ‘Regulation of actin cytoskeleton’, ‘Tight junction’, ‘Adherens junction’ and ‘Oxytocin signaling pathway’. Metascape analysis identified Reactome gene sets including ‘Membrane Trafficking’, ‘EPH-Ephrin signaling’, ‘RHO GTPases activate PAKs’, ‘Gap junction degradation’, ‘Smooth Muscle Contraction’, and ‘Cell-Cell communication’, and CORUM protein complexes including ‘Emerin complex 1' (Table II).

**Table II.** Gene Set Enrichment Analysis for PPP1CB target genes

**A,CORUM**					
Term	Description	Count	Frequency,%	Log10(P)	Log10(q)
CORUM:5604	Emerin complex 1	5	2.26	−8.79	−6.42
**B, GO Biological Processes**					
Term	Description	Count	Frequency,%	Log10(P)	Log10(q)
GO:0030036	actin cytoskeleton organization	70	31.67	−56.1	−51.75
GO:0035303	regulation of dephosphorylation	26	11.76	−22.85	−19.65
GO:0030048	actin filament-based movement	19	8.6	−17.05	−14.12
GO:0010638	positive regulation of organelle organization	29	13.12	−13.43	−10.75
GO:0031032	actomyosin structure organization	18	8.14	−13.39	−10.72
GO:0000904	cell morphogenesis involved in differentiation	30	13.57	−12.45	−9.84
GO:0051301	cell division	27	12.22	−12.4	−9.8
GO:0034329	cell junction assembly	18	8.14	−11.54	−9
GO:0051017	actin filament bundle assembly	14	6.33	−10.39	−7.93
GO:0051014	actin filament severing	6	2.71	−8.77	−6.41
**C, KEGG Pathway**					
Term	Description	Count	Frequency,%	Log10(P)	Log10(q)
ko04810	Regulation of actin cytoskeleton	20	9.05	−15.27	−12.46
hsa04530	Tight junction	17	7.69	−13.1	−10.46
ko04921	Oxytocin signaling pathway	14	6.33	−10.7	−8.18
ko04520	Adherens junction	9	4.07	−8.25	−5.92
**D,Reactome Gene Sets**					
Term	Description	Count	Frequency,%	Log10(P)	Log10(q)
R-HSA-199,991	Membrane Trafficking	33	14.93	−17.03	−14.12
R-HSA-2,682,334	EPH-Ephrin signaling	15	6.79	−15.16	−12.37
R-HSA-5,627,123	RHO GTPases activate PAKs	9	4.07	−12.92	−10.3
R-HSA-190,873	Gap junction degradation	6	2.71	−9.7	−7.27
R-HSA-445,355	Smooth Muscle Contraction	8	3.62	−9.08	−6.68

CORUM: The comprehensive resource of mammalian protein complexes. KEGG: Kyoto Encyclopedia of Genes and Genomes; q: false positive rate.

#### Identification of node genes through PPI network analyses

**Figure 3.**
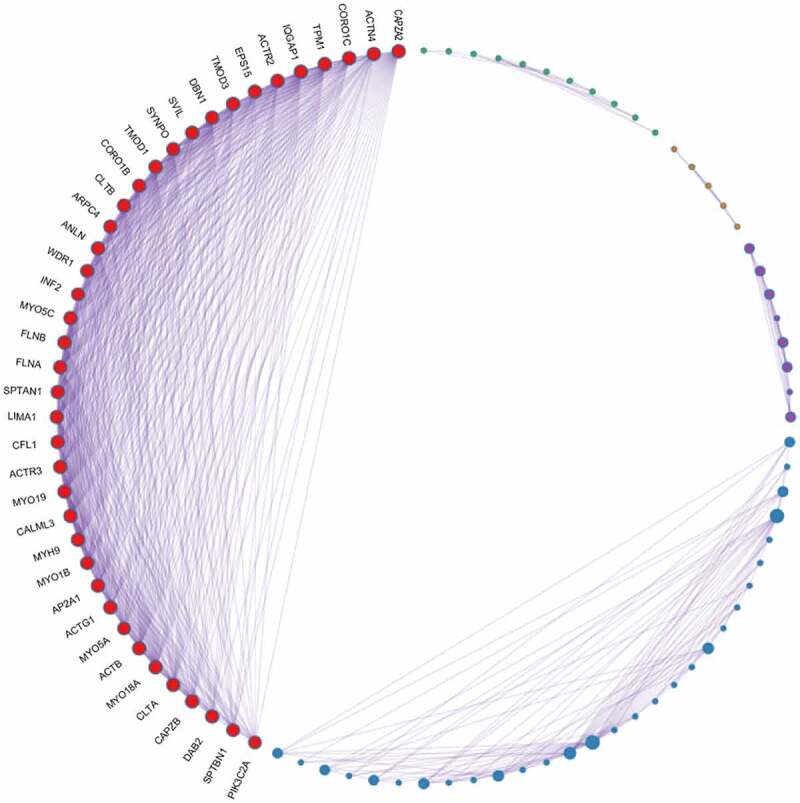
PPI network analysis of overlapping PPP1CB target genes. The PPI network contained 222 genes. Different nodes represent different genes and corresponding proteins connected in the PPI network. A variety of genes cooperate with each other to carry out biological functions. Red nodes indicate a node score of 13.64, blue nodes indicate a node score of 2.97, purple nodes indicate a node score of 2.875, orange nodes indicate a node score of 1.6, and green nodes indicate a node score of 1.273. Densely connected network components were identified via the MCODE algorithm, and the 39 genes with the highest score were selected as node genes (node score 13.64). PPI: protein protein interaction

PPI network analyses was performed using Metascape software (Figure 3) to further study the relationship between the 222 overlapping target genes. The MCODE algorithm was used to identify dense networks and to connect each adjacent MCODE component with different colors to represent different types of biological interactions. We identified 39 node genes: *ACTB, ACTG1, ACTN4, ACTR2, ACTR3, ANLN, AP2A1, ARPC4, CALML3, CAPZA2, CAPZB, CFL1, CLTA, CLTB, CORO1B, CORO1C, DAB2, DBN1, EPS15, FLNA, FLNB, INF2, IQGAP1, LIMA1, MYH9, MYO18A, MYO19, MYO1B, MYO5A, MYO5C, PIK3C2A, SPTAN1, SPTBN1, SVIL, SYNPO, TMOD1, TMOD3, TPM1*, and *WDR1.*

#### Node genes are differentially expressed between pancreatic adenocarcinoma and matched paracancerous tissue and may have prognostic value for PAAD outcomes

**Figure 4.**
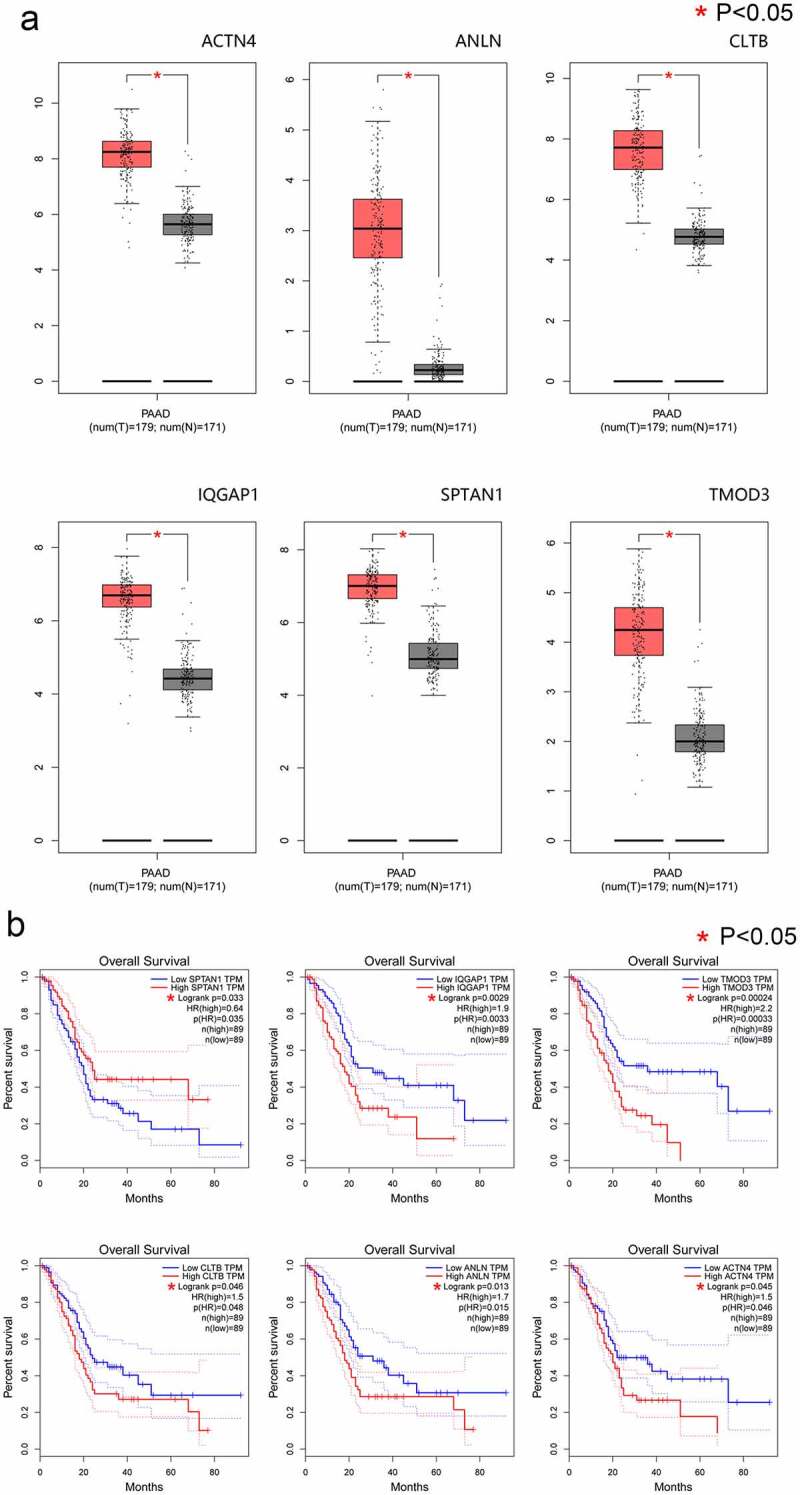
Evaluation of gene expression and prognostic significance of six nodal *PPP1CB* gene targets in PAAD. (a) Expression levels of six selected nodal genes were evaluated in PAAD and matched paracancerous tissues. The expression of 6 nodal genes in PAAD tissues (n = 179) was significantly higher compared with the matched paracancerous tissues (n = 171). Red and gray indicate PAAD and matched paracancerous tissues, respectively. (b) Prognostic value for the six nodal genes related to overall survival for patients with PAAD. Kaplan-Meier curves were generated using an online tool (data from the TCGA dataset). PAAD: pancreatic cancer. *P < 0.05.

We used the GEPIA tool to analyze expression of the 39 node genes in PAAD tissues and matched paracancerous tissues (Supplementary file 2). The expression of *ACTB, ACTG1, ACTN4, ACTR2, ACTR3, ANLN, AP2A1, ARPC4, CAPZA2, CAPZB, CFL1, CLTA, CLTB, CORO1B, CORO1C, DAB2, DBN1, EPS15, FLNA, FLNB, INF2, IQGAP1, LIMA1, MYH9, MYO18A, MYO5A, SPTAN1, SPTBN1, SVIL, SYNPO, TMOD3, TPM1*, and *WDR1* were significantly increased in PAAD tissue (*P < 0.05*) (Supplementary File 3). The GEPIA RNA-SEQ dataset is based on the UCSC Xena project and is analyzed through a standard pipeline (http://xena.ucsc.edu) [19]. The GEPIA approach solves the problem of invalid analysis resulting from differences between different tumor types. Furthermore, the overall survival rate of PAAD cases with high expression of six nodal genes, *ACTN4, ANLN, CLTB, IQGAP1, SPTAN1*, and *TMOD3*, was significantly lower than those expressing low levels of these nodal genes. (Figure 4)

#### SiRNA knockdown of PPP1CB expression in PAAD cells

**Figure 5.**
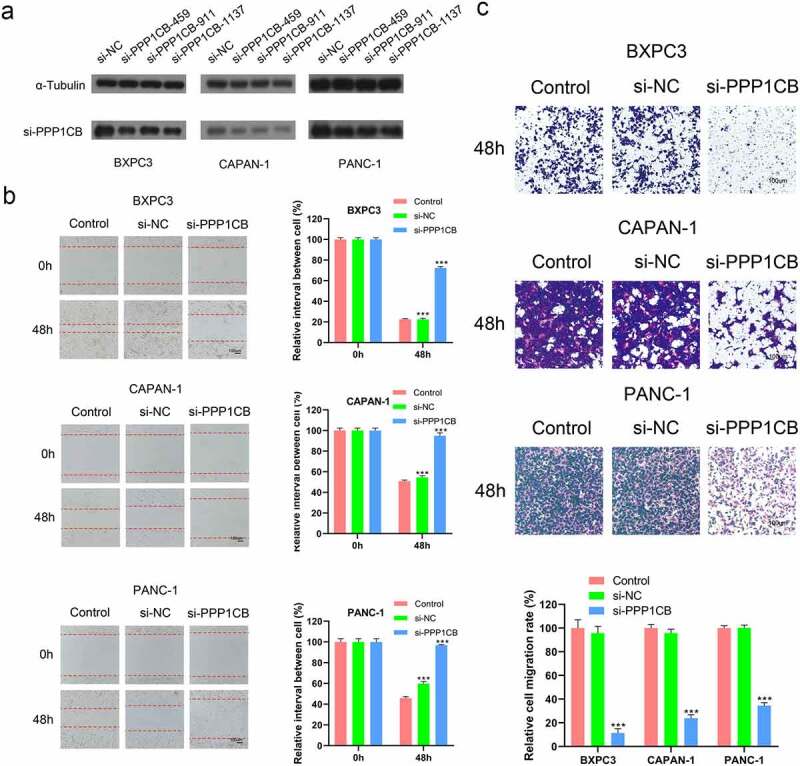
Effects of *PPP1CB* knockdown on invasion and migration of PAAD cell lines. (a) *PPP1CB* was knocked down in BXPC3, CAPAN-1, and PANC-1 cells using siRNA: si-NC (non-silencing control), si-*PPP1CB*-459, si-*PPP1CB*-911 and si-*PPP1CB*-1137. Knockdown efficiency was evaluated by Western Blot. (b) *PPP1CB* knockdown reduces PAAD cell migration. The wound healing assay was performed in BXPC3, CAPAN-1, and PANC-1 cell monolayers for 48 h, and the width of the gap for each group at 0 hours was used as the reference. (c) Invasion analyses of PAAD cells transfected with si-NC, or si-*PPP1CB*, relative to the invasion in the control group, reported as mean ± SD (n = 3). ***P < 0.001.

We knocked down the expression of *PPP1CB* in PAAD cells and verified the knockdown of *PPP1CB* protein by Western Blot. All three of the siRNA constructs (si-*PPP1CB*-459, si-*PPP1CB*-911, and si-*PPP1CB*-1137) significantly inhibited *PPP1CB* expression in BXPC3, CAPAN-1, and PANC-1 cells; si-*PPP1CB*-1137 achieved the most significant down-regulation of *PPP1CB*, and was selected to knock down *PPP1CB* in subsequent experiments (Figure 5a).

#### PPP1CB facilitates the migration of PAAD cells

In order to study whether *PPP1CB* might contribute to PAAD metastasis, we used si-*PPP1CB*-1137 to knockdown *PPP1CB* expression in BXPC3, CAPAN-1, and PANC-1 cells. After transfection, the expression of *PPP1CB* in cells decreased and cell migration was inhibited. PAAD cell migration was significantly inhibited by si-*PPP1CB* 48 h after transfection, compared with the control group and the si-NC group. However, there was no significant difference in migration between the control group and the si-NC group. These results indicate that *PPP1CB* promotes the migration of PAAD cells ***in vitro*** (Figure 5b).

#### Effects of PPP1CB silencing on the invasion of PAAD cells in vitro

Transwell invasion assays were performed to evaluate the influence of *PPP1CB* knockdown on the invasion capability of BXPC3, CAPAN-1, and PANC-1 cells. Compared to the control group, the si-*PPP1CB* group exhibited significantly reduced cell invasion, while there was no difference between the control and si-NC group. These results demonstrate that *PPP1CB* knockdown inhibits the invasion of PAAD cells *in vitro* (Figure 5c)

#### PPP1CB expression in PAAD clinical samples

**Figure 6.**
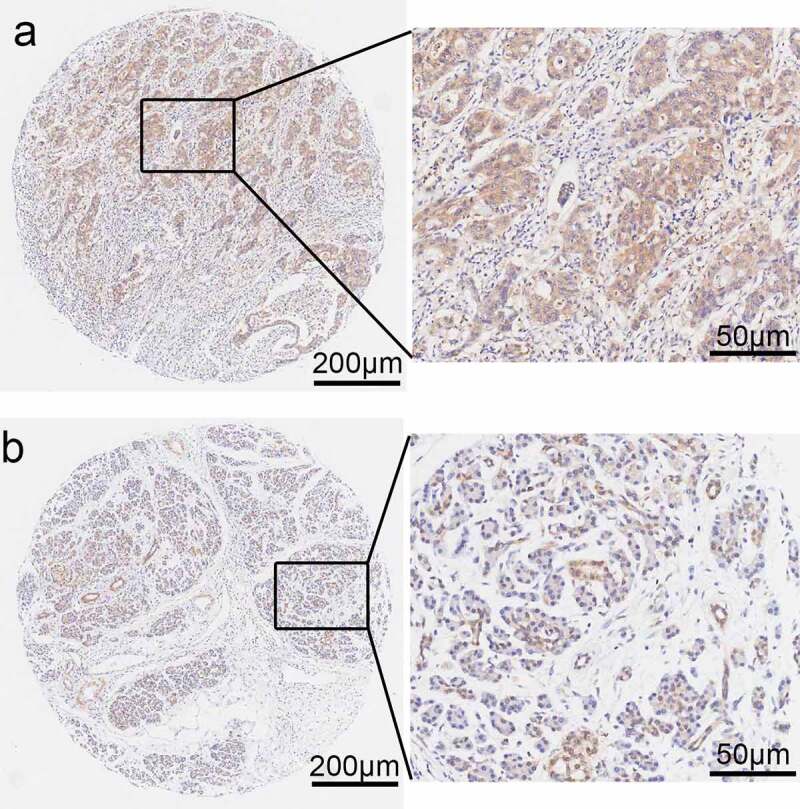
Photomicrographs of immunohistochemical staining for *PPP1CB* in PAAD clinical patient tissue samples. (a) Positive staining for *PPP1CB*. (b) Negative staining for PPP1CB. PPP1CB: protein phosphatase 1 catalytic subunit beta

Immunohistochemical staining was performed to determine the expression levels of *PPP1CB* in 88 PAAD samples and matched paracancerous tissue samples (Figure 6). *PPP1CB* expression was high in 50 PAAD tissue samples (56.82%) and low in 38 samples (43.18%). However, in the matched paracancerous tissues, *PPP1CB* expression was high in 34 samples (38.64%) and low in 54 samples (61.36%). Compared with the matched paracancerous tissue samples, *PPP1CB* expression in PAAD tissue was significantly increased (*P = 0.016*; Table III).

**Table III.** The expression levels of PPP1CB in clinical PAAD tissue are significantly higher compared with matched paracancerous tissue samples

		*PPP1CB* expression		χ2	
Tissue type	n	Low (%)	High (%)		*p value*
Pancreatic cancer	88	38	50	5.830	*0.016*
Paracancerous tissues	88	54	34		

n = 88 in each group. PPP1CB: Protein phosphatase 1 catalytic subunit beta; PAAD: pancreatic adenocarcinoma.

#### PPP1CB expression levels are associated with clinicopathological characteristics

*PPP1CB* expression levels were found to be associated with sex (*P = 0.048*), alcohol use (*P = 0.039*), CEA (*P = 0.038*), N stage (*P = 0.001*), and invasion of nerve status (*P = 0.036*). *PPP1CB* expression was not significantly associated with other clinicopathological characteristics (Table IV).

**Table IV.** Relationship between PPP1CB expression and clinicopathological characteristics in PAAD patients

Clinicopathoogical characteristics	variables	*PPP1CB* expression		total	χ^2^	*p value*
		low	high			
Age (years)					0.078	*0.781*
	≤60	20	24	44		
	>60	20	27	47		
Sex					3.899	*0.048*
	Female	19	14	33		
	Male	21	37	58		
Grade					2.214	*0.137*
	I/II	32	35	67		
	III	4	11	15		
	No data			9		
T stage					0.065	*0.799*
	T0-2	13	15	28		
	T3-4	27	35	62		
	No data			1		
Jaundice					0	*0.984*
	Negative	13	18	31		
	Positive	10	14	24		
	No data			36		
Alcohol use					4.275	*0.039*
	NO	35	36	71		
	YES	4	14	18		
	No data			2		
Tumor site					3.202	*0.074*
	Head	20	33	53		
	Other	20	15	35		
	No data			3		
CA199					1.849	*0.174*
	Negative	6	13	19		
	Positive	32	33	65		
	No data			7		
CEA					4.293	*0.038*
	Negative	22	37	59		
	Positive	16	10	26		
	No data			6		
Tumor sizes (cm)					0.002	*0.969*
	≤5	21	25	46		
	>5	19	23	42		
	No data			3		
N stage					11.025	*0.001*
	N0	30	20	50		
	N1	10	30	40		
	No data			1		
M stage					NA	0.501*
	M0	40	48	88		
	M1	0	2	2		
	No data			1		
TNM stage					0.619	*0.431*
	I–II	38	44	82		
	III–IV	2	6	8		
	No data			1		
Histology					0	*1*
	AD	31	36	67		
	ASC	4	4	8		
	No data			16		
Invasion of nerve					4.38	*0.036*
	NO	21	15	36		
	YES	19	34	53		
	No data			2		
Invasion of large blood vessels					3.673	*0.055*
	NO	32	30	62		
	YES	8	19	27		
	No data			2		
p53					0.109	*0.741*
	Negative	21	24	45		
	Positive	19	25	44		
	No data			2		
TopoII					0.013	*0.908*
	Negative	8	8	16		
	Positive	30	32	62		
	No data			13		
Diabetes					0.686	*0.407*
	NO	6	12	18		
	YES	8	9	17		
	No data			56		
Smoking					3.094	*0.079*
	NO	33	33	66		
	YES	7	17	24		
	No data			1		
Ki67					NA	0.455*
	Negative	1	0	1		
	Positive	39	48	87		
	No data			3		
P63					NA	1*
	Negative	25	32	57		
	Positive	0	1	1		
	No data			33		

*Fisher’s exact test. PPP1CB: Protein phosphatase 1 catalytic subunit beta; NA: not applicable.

#### Relationship between the expression of PPP1CB and overall survival

**Figure 7.**
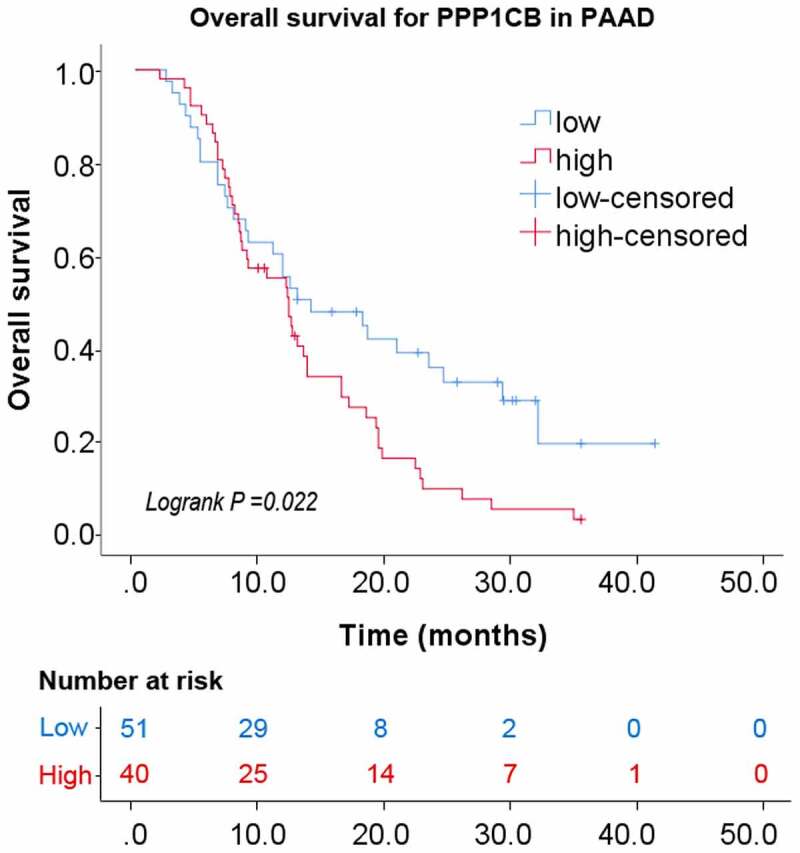
Kaplan-Meier analysis of PAAD. *PPP1CB* protein expression impact on overall survival. Immunohistochemical staining of PAAD tissue microarrays was used to determine the expression levels of *PPP1CB. PPP1CB*: protein phosphatase 1 catalytic subunit beta; PAAD: pancreatic adenocarcinoma

Kaplan–Meier analysis with a log-rank test was performed for univariate survival analyses. The overall survival of the *PPP1CB* high-expression group was significantly reduced than the *PPP1CB* low-expression group (*P = 0.022*; Figure 7). The results demonstrate a correlation between high *PPP1CB* expression and poor prognosis in PAAD, and suggest that *PPP1CB* might have value as an independent prognostic factor for PAAD outcomes.

## Discussion

Pancreatic cancer (PAAD) is one of the main causes of cancer-related deaths especially in more developed countries [3]. PAAD often lacks distinct symptoms at early stages, and the prognosis of PAAD is usually poor, with a 5-year survival rate of merely 8% ^[1]^. Surgery is the only curative treatment for PAAD, and immunotherapy, chemotherapy and radiotherapy are only used to improve long-term survival as auxiliary therapies [28]. The discovery of novel therapeutic targets is critical in the development of tumor-specific targeted treatments. New treatments have emerged in recent years, including targeted therapies that target genes involved in the pathophysiological processes of PAAD [29]. Zhu *et al*. reported that reducing the activity of *PPP1CB* inhibits the progression of prostate cancer [30]. Additionally, it has been shown that *PPP1CB* is related to tumor cell migration and metastasis, *PPP1CB* can promote actomyosin contraction and accelerate tumor metastasis when the *PPP1CB* inhibitor is phosphorylated [9]. In this study, we conducted a bioinformatics analysis using the TCGA dataset to understand whether *PPP1CB* is related to the prognosis of PAAD, and validated the results in cell experiments *in vitro* and in clinical tissue samples from patients with PAAD. Our findings support the hypothesis that *PPP1CB* facilitates the progression of PAAD. The expression of PPP1CB in PAAD tissue was notably higher than in matched paracancerous tissues from patient samples. Experiments *in vitro* demonstrated that knockdown of *PPP1CB* inhibited the migration and invasion capability of PAAD cell lines. In addition, immunohistochemical staining of clinical tissue samples show that high *PPP1CB* expression was associated with sex, alcohol use, CEA, N stage and invasion of nerve. Survival analysis suggested that the overall survival rate of PAAD patients with low *PPP1CB* expression was better than patients with high *PPP1CB* expression. These data demonstrate that the expression levels of *PPP1CB* in PAAD tissue may have prognostic significance in predicting outcomes for patients with PAAD. In recent years, it has been reported that *PPP1CA, PPP1CB, PPP2CA, PPP2CB*, and *PPP3CA* accelerate the progression of pancreatic cancer, while *PPP3CB, PPP5C*, and *PPP6C* hinder PAAD progression [16] .

In addition, studies have reported that *PPP1CB* has a positive relationship with the proliferation, migration and invasion of prostate cancer, gastric cancer, and melanoma, however studies have not evaluated the specific role of *PPP1CB* [13,14,30]. In this study, the expression and prognosis of *PPP1CB* in PAAD was evaluated through bioinformatics analysis, and the relationship between *PPP1CB* expression, and migration and invasion ability was verified at the cellular level. Finally, immunohistochemical analysis of *PPP1CB* expression in clinical PAAD samples was used to determine whether *PPP1CB* can be used as an independent prognostic factor for PAAD. However, when exploring the relationship with *PPP1CB* expression level and clinicopathological characteristics, incomplete clinicopathological data collection may confound the results. Furthermore, the preponderance of Chinese patients represented in the PAAD and matched paracancerous tissue comparison necessitates that these results should be further verified in other ethnically diverse populations. In addition, the precise mechanism by which *PPP1CB* contributes to poor outcome of PAAD requires further evaluation.

## Conclusion

In conclusion, our data shows that *PPP1CB* may serve as an independent predictor of poor outcome, and *PPP1CB* has the potential to be used for early diagnosis of PAAD. Following further investigation, this could improve the survival of patients with pancreatic cancer.

## Supplementary Material

Supplemental Material

## Data Availability

The datasets used and/or analyzed during the current study are available from the corresponding author upon reasonable request.

## Highlight

1. High *PPP1CB* expression is associated with poor prognosis in patients with PAAD in TCGA
2. *PPP1CB* promoted the migration and invasion capability of PAAD cell lines.
3. *PPP1CB* expression levels are associated with clinicopathological characteristics.
4. *PPP1CB* may serve as an independent predictor of poor outcome.
