# Supplementary material for: The expression and clinical prognostic value of protein phosphatase 1 catalytic subunit beta in pancreatic cancer
Source: Bioengineered. 2021 Jun 14;12(1):2763–78. doi: 10.1080/21655979.2021.1934243 (PMC8806868; doi:10.1080/21655979.2021.1934243)
Supplement: Supplemental Material [file KBIE_A_1934243_SM8003.zip › Supplementary file 1.docx]

Supplementary file 1. Pancreatic adenocarcinoma‑associated genes identified in The Cancer Genome Atlas using five databases (data from TCGA datasets).

| **Database Gene name** |
| --- |
| **InBio Map**  YLPM1，SHOC2，PPP1R3B，PPP1CC，TOX4，PPP1CA，PPP1CB，AKAP11，MYH14，ATIC，ROCK1，SFRP5，PAPOLG，CPSF2，WBP11，DNAAF2，PPP1R1A，PPP1R12C，KIF14，PPP1R11，ROCK2，MYL9，PPP1R15A，SSU72，PPP1R3A，PPP1R8，LZTR1，PPP1R2，PPP1R10，PPP1R3C，PAPOLB，PPP1R12B，MYL6，PLIN1，PPP1R9A，WDR33，NUAK1，PPP1R16A，SH2D4A，MYL12B，RAI14，PPP1R16B，ZFYVE9，TRIOBP，PPP1R12A，CCDC85C，MYH9，PPP1R9B，MYH11，WDR82，SFRP2  **STRING** INTS6，INTS1，PPP1R12，PPP1R7，PPP1CC，PPP1CA，PPP1R11，PPP1R2，WDR82，PPP1R12A  **BioGRID** PPP1R7、PPP1R12A、PPP1R2、PPP1R11、PPP1R8、SH2D4A、TMEM33，NCL，AKAP11，PPP1R15A，SMARCB1，PPP1R9B，RYR2，PTK2，PPP1R12B，BRCA1，NCOR1，TRIM28，HDAC7，INTS1，INTS6，WDR82，TOX4，PPP1R10，UBC，Cep72，Sass6，NONO，ELAVL1，SIRT7，BCL2，LBR，CDKN2A，CDC34，CCNB1，CCND1，CCND3，HSPA4，MAX，CUL3，AURKA，PPP1CA，PAPOLA，OGT，HCFC1，KAT8，WDR5，MDM4，RHO，ZFYVE9，PAN2，DHX15，MVK，DENR，EEF1A1，NUAK1，PPP1R3C，TP53BP2，CSRNP2，PPP1R16A，PPP1R2P3，EED，AKT1，LUZP4，ABCE1，CSNK2B，1-Dec，CLOCK，RORC，PPP1R18，PPP1R13B，PPP1R3A，RASSF7，RASSF8，RASSF9，UNK，DAG1，CPSF1，PPP1CC，SSU72，NTRK1，MED23，CLK1，ACTA1，ACTB，ACTG1，ACTN4，ACTN1，AP2A1，ANXA2，DST，CALM1，CALML3，CAPZA1，CAPZA2，APZB，CFL1，CFL2，CLTA，CLTB，DAB2，DAPK3，DBN1，DDX3X，DHX9，CTTN，EPS15，FLII，FLNA，FLNB，HNRNPA2B1，HNRNPH3，ILF3，ABLIM1，LMO7，MYO1B，MYLK，MYO1C，MYO1E，MYO5A，MYO5B，MYO6，PIK3C2A，PLEC，CDK2，MYH9，IQGAP1，MATR3，SYNPO，ASF1A，ANLN，MYO18A，TWF1，SAFB，SIPA1，SPTAN1，SPTBN1，SPTBN2，SSFA2，ST5，SVIL，TJP1，TMOD1，TNFAIP1，TPM1，TPM2，TPM3，TPM4，YES1，LUZP1，TAF15，SORBS2，URI1，APOBEC3B，SAFB2，CLINT1，ARHGAP11A，SEC16A，WDR1，ARPC4，ACTR3，ACTR2，ARPC2，RBM6，BASP1，GAS2L1，KHDRBS1，CCT8，PPP1R13L，DSTN，TRIOBP，AKAP2，SIRT2，LIMCH1，SIPA1L3，MPRIP，COBL，SPECC1L，CORO1C，ZDHHC5，PLEKHG3，RAI14，TES，RBMX，ZNF638，RACGAP1，TMOD3，LIMA1，PPP1R12C，PIH1D1，UACA，RIF1，BMP2K，MYO5C，YLPM1，CORO1B，ARHGAP21，NCOA5，AFAP1，ZNF106，INF2，METTL17，MYO19，PDRG1，PPP1R15B，KIAA1671，STON2，SSH2，NEXN，SPECC1，WDR92，MISP，ACTRT1，KIF18B，CDCA2，UBXN2A，GAS2L3，ZNF326，TPRN，RBM12B，RBMXL1，Itga5，Myh10，Arrb2，FBXW7，GLI1，USP37，UBXN2B，UBXN1，ACTN2，CCDC85C，GSK3A，GSK3B，LRRC1，PLCL2，SCRIB，SHOC2，PPP1R16B，LMTK2，AATK，SFRP2，SFRP1，SFRP5，RRP1B，C20orf27，KIF18A，POLR2E，WBP11，TLK1，UXT，PQBP1，RPAP3，PACS1，DCAF7，DYNLL2，INTU，PPP2R5C，CD2BP2，TRIM25，ORF23，HNRNPL，FBXO7，TRIM14，ARMC8，HIST1H3A，H3F3A，PAK2，PPP1R37，GYS1，MINPP1，KPNB1，WDR77，PPP6C，ACO2，CTNNB1，YAF2，RAD18，EFTUD2，RPTOR，TNIP2，CHD3，CHD4，TNF，GNB1，FGF11，ESR2，LARP7，RNF144A，REST，MYC，Prkaa1，Prkab1，HIST1H4A，HIST1H2AB，NEK2，GRWD1，ATG16L1，Bach1，ZBTB10，LZTR1，PRDM16，YAP1，MAPT，VRK3，ANO7，PGRMC1，WWP2，PHACTR3，PPP1R2P9，MEOX2，CHCHD3，CSRNP1，SPATC1L，PPP1R27，C14orf180，CYSRT1，KANK2，PLEKHA4，HNRNPD，ORF9b，HOXA2，UBAC1，nsp8，nsp10，nsp13，  **IntAct**  SH2D4A，PPP1R7，PPP1R11，PPP1R13L，PPP1R16A，PPP1R12A，PPP1R3C，PPP1R16B，CSRNP2，PPP1R2B，PPP1R8，RRP1B，CCDC85C，RIF1，URI1，PPP1CA，WDR92，PPP1R2，PPP1R12B，TOX4，TP53BP2，PPP1R13B，PPP1R10，UACA，BRCA1，CYSRT1，PPP1R2C，ACTN2，KANK2，MEOX2，PPP1R27，NRAC，PHACTR3，CSRNP1，PPP1R9B，SPATC1L，CHCHD3，PPP1R9A，Clock，PQBP1，Rpl5，POLR2E，DCAF7，KIF18A，DYNLL2，C20orf27，RPAP3，UXT，TLK1，WBP11，ORF23，PPP1R37，NCL，YLPM1，KPNB1，WDR82，PPP1R12C，TRIOBP，RAI14，MYL6，Ptk2，RB1，BCL2，CCND1，Max，CCND3，CDC34，TMEM33，TPRN，Sass6，CDKN2A，Cep72，RIPK3，IKBKG，trxB，BHLHE40，ZFYVE9，PCNA，RORC，CSNK2B，PPP2R5C，DUX4L9，AATK，PPP1R3B，LMTK2，thiC，PLEKHG3，Prdm16，KHC，pi3p，e9peb9，TJP1，GAS2L3，MPRIP，TPM1，YES1，ACTG1，PIK3C2A，DDX3X，ABLIM1，SPTBN2，SEC16A，ARPC2，Smad6，DAPK3，ACTN4，MYO1B，SIPA1L3，WDR1，COBL，FLNB，PPP1R15A，RASSF9，SORBS2，SVIL，AP2A1，NS，JUN，GSN，TPM3，ANXA2，TPM2，CLTA，CLTB，CALM1，rep，Itga5，ACTN1，GYS1，CD59，Mecom，JUP，Tnf，VCAM1，Slc2a4，FLNA，HNRNPA2B1，CFL1，CDK2，CALML3，TMOD1，ITPRID2，HNRNPH3，Rab21，MYH9，PPP1CC，RBMX，MNDA，EPS15，MATR3，Nf2，IQGAP1，CAPZA2，CAPZB，CCT8，Cav3，CAPZA1，Prkaa1，ARPC4，ACTB，DSTN，ACTR3，ACTR2，GRB2，TPM4，ACTC1，UBXN2A，RBM6，DENND2B，Prkab1，BASP1，DAB2，SPTBN1，RASSF7，KHDRBS1，DHX9，NEXN，TWF1，ILF3，MYO1E，ECH1，FLII，TUBB3，CUL3，SPTAN1，TNFAIP1，SAFB2，CTTN，CLINT1，ZNF638，PLEC，SAFB，MYLK，DBN1，INF2，ZNF326，SPECC1，PPP1R15B，ARHGAP21，L1TD1，Myh10，CDCA2，SPECC1L，PLEKHA7，PPP1R18，ARHGAP11A，TUBA1A，SSH2，LUZP1，KIF18B，Taf15，MISP，SIRT2，RBM12B，SYNPO，AFAP1，RASSF8，ACTRT1，LMO7，STON2，Arrb2，MYO18A，RBMXL1，SIPA1，MYO19，GAS2L1，CORO1B，KIAA1671，ZDHHC5，RACGAP1，ZNF106，EPB41L1，METTL17，NCOA5，ANLN，MYO5C，BMP2K，PDRG1，PIH1D1，TMOD3，TES，APOBEC3B，LIMA1，INTU，MYO5B，CORO1C，MYO6，MINPP1，LIMCH1，Myo1c，CFL2，ASF1A，AKAP2，MYO5A，Ywhae，CLTC，AHNAK，PABPC4，DSG2，SCRIB，AAK1，Agap2，Dlg4，Mycbp2，PARD3，AP2M1，Ywhab，Xpo7，ANTXR1，PACSIN3，LRRFIP2，LRRK2，PACS1  **HPRD**  PTK2，PRKAR2A，PPP1R12B，RB1，ZFYVE9，WBP11，PPP1R12C，TLX1，CDKN1B，PPP1CA，PPP1CC |
