# Supplementary figures and images for: The expression and clinical prognostic value of protein phosphatase 1 catalytic subunit beta in pancreatic cancer
Source: Bioengineered. 2021 Jun 14;12(1):2763–78. doi: 10.1080/21655979.2021.1934243 (PMC8806868; doi:10.1080/21655979.2021.1934243)

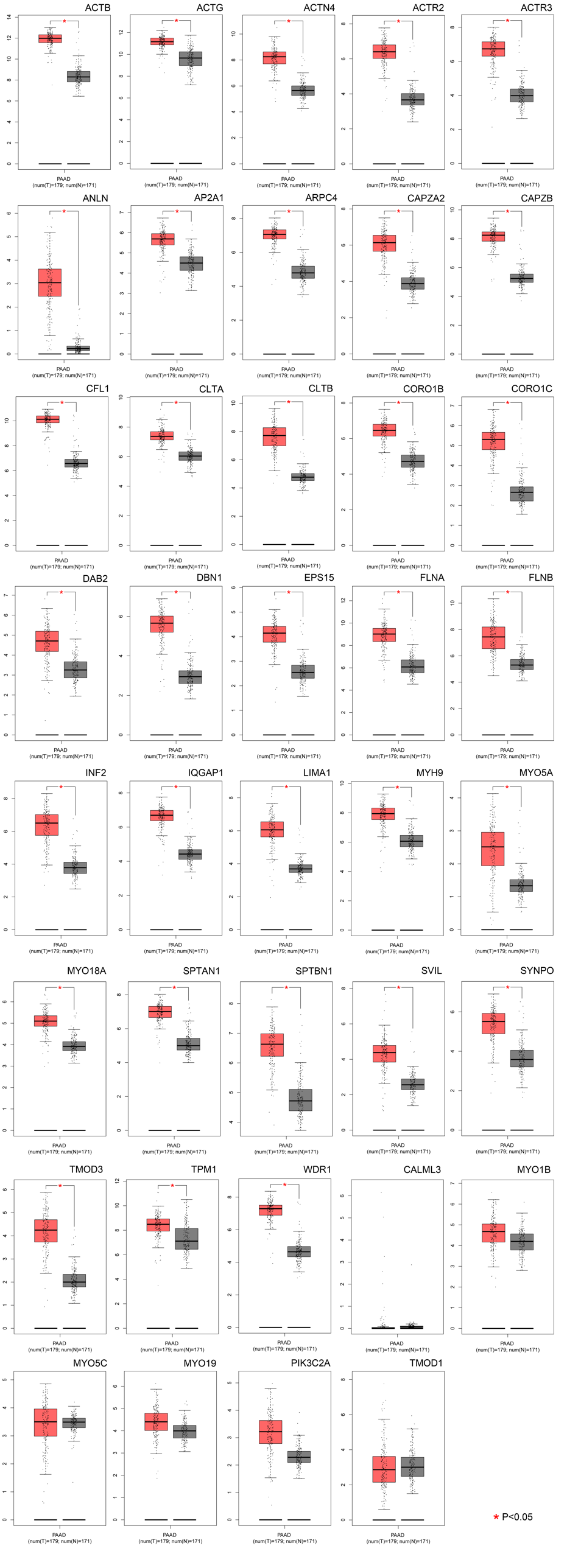

Supplement: Supplemental Material [file KBIE_A_1934243_SM8003.zip › Supplementary file 2.tif]
